# Optimizing diagnosis and surgical decisions for chronic osteomyelitis through radiomics in the precision medicine era

**DOI:** 10.3389/fbioe.2024.1315398

**Published:** 2024-05-10

**Authors:** Qiyu Jia, Hao Zheng, Jie Lin, Jian Guo, Sijia Fan, Abudusalamu Alimujiang, Xi Wang, Lanqi Fu, Zengru Xie, Chuang Ma, Junna Wang

**Affiliations:** ^1^ The First Affiliated Hospital of Xinjiang Medical University, Urumqi, China; ^2^ The First Affiliated Hospital of Zhejiang Chinese Medical University (Zhejiang Provincial Hospital of Chinese Medicine), Hangzhou, China

**Keywords:** chronic osteomyelitis, radiomics, MRI, diagnostic accuracy, surgical decisionmaking, shape features, region of interest

## Abstract

**Introduction:** Chronic osteomyelitis is a complex clinical condition that is associated with a high recurrence rate. Traditional surgical interventions often face challenges in achieving a balance between thorough debridement and managing resultant bone defects. Radiomics is an emerging technique that extracts quantitative features from medical images to reveal pathological information imperceptible to the naked eye. This study aims to investigate the potential of radiomics in optimizing osteomyelitis diagnosis and surgical treatment.

**Methods:** Magnetic resonance imaging (MRI) scans of 93 suspected osteomyelitis patients were analyzed. Radiomics features were extracted from the original lesion region of interest (ROI) and an expanded ROI delineated by enlarging the original by 5 mm. Feature selection was performed and support vector machine (SVM) models were developed using the two ROI datasets. To assess the diagnostic efficacy of the established models, we conducted receiver operating characteristic (ROC) curve analysis, employing histopathological results as the reference standard. The model’s performance was evaluated by calculating the area under the curve (AUC), sensitivity, specificity, and accuracy. Discrepancies in the ROC between the two models were evaluated using the DeLong method. All statistical analyses were carried out using Python, and a significance threshold of *p* < 0.05 was employed to determine statistical significance.

**Results and Discussion:** A total of 1,037 radiomics features were extracted from each ROI. The expanded ROI model achieved significantly higher accuracy (0.894 vs. 0.821), sensitivity (0.947 vs. 0.857), specificity (0.842 vs. 0.785) and AUC (0.920 vs. 0.859) than the original ROI model. Key discriminative features included shape metrics and wavelet-filtered texture features. Radiomics analysis of MRI exhibits promising clinical translational potential in enhancing the diagnosis of chronic osteomyelitis by accurately delineating lesions and identifying surgical margins. The inclusion of an expanded ROI that encompasses perilesional tissue significantly improves diagnostic performance compared to solely focusing on the lesions. This study provides clinicians with a more precise and effective tool for diagnosis and surgical decision-making, ultimately leading to improved outcomes in this patient population.

## 1 Introduction

Chronic osteomyelitis has long been recognized as one of the most challenging diseases in the medical field, often referred to as the “second cancer” (Zhang et al., 2019; Masters et al., 2022). Despite the refined antimicrobial activity of new-generation antibiotics and the efficacy of surgical intervention, the recurrence rate of chronic osteomyelitis remains as high as 20%–30% (Conterno and Turchi, 2013; Chastain and Davis, 2019; Zeitlinger, 2019; Wang X. et al., 2023).

Accurate diagnosis of chronic osteomyelitis is essential, as misdiagnosis can lead to the worst outcomes due to differences in treatment approaches. Magnetic Resonance imaging (MRI), with its excellent contrast between bone and soft tissue, is currently one of the most valuable tools for diagnosing chronic osteomyelitis. However, relying solely on MRI can be challenging for differentiating diseases with similar radiographic features, such as bone tuberculosis and osteosarcoma. These conditions often present with bone marrow edema, soft tissue masses, and inflammatory changes, which may overlap in imaging characteristics, thus complicating differential diagnosis and potentially affecting subsequent treatment strategies. Furthermore, once a definitive diagnosis is made, thorough debridement is necessary, as incomplete debridement can lead to recurrent infections (Bosse et al., 2002). Therefore, relying on intraoperative judgment based on experience, experienced orthopedic surgeons have increasingly adopted expanding debridement as the preferred approach. However, this practice can result in extensive bone defects, which pose a significant challenge during the postoperative period (Heng et al., 2023). On the other hand, narrowing the debridement area increases the risk of infection recurrence. Thus, it seems challenging to strike a balance between the two approaches. Moreover, even with the expansion of the debridement area, there are instances where chronic osteomyelitis still recurs, highlighting the limitations of current interventional approaches and the harsh reality faced in clinical practice (Hotchen et al., 2020).

Radiomics, an emerging diagnostic and adjunctive imaging technique, has witnessed rapid development in recent years, offering hope in addressing this issue (Huang et al., 2016; Witt et al., 2020; Bera et al., 2022; Wang W. et al., 2023). High throughput radiomics transforms traditional medical images into highly reliable, reproducible, and non-redundant data that can be mined for valuable information by extracting and analyzing a large volume of advanced and quantitative image feature data. These extracted features provide insights into pathological and physiological phenomena that are not readily discernible by the naked eye in chronic osteomyelitis, particularly in terms of structural damage and alterations in image texture (Gillies et al., 2015; Huang et al., 2016; Bera et al., 2022; Cuce et al., 2023). Currently, this technique is primarily applied in the classification and prediction of various cancers (Lambin et al., 2017; Hassani et al., 2019). Its high diagnostic accuracy can also be leveraged to reduce the misdiagnosis rate of chronic osteomyelitis by mining big data from radiographic images and facilitating early diagnosis and treatment.

For orthopedic surgeons, the future potential primary advantage of this technique lies in optimizing surgical decision-making. The infected area and its extent can be accurately determined by analyzing preoperative imaging data and establishing precise three-dimensional reconstruction images combined with machine learning and image segmentation techniques. The development of expanded detection technology provides the foothold for optimal debridement in cases of osteomyelitis (Wu et al., 2021), which involves improving the discrimination of the primary lesion area and defining a reliable and safe zone based on “artificial intelligence” judgments. This approach can assist in preoperative planning and optimizing debridement strategies, allowing surgeons to treat patients more accurately and efficiently while ensuring complete removal of infected tissue and minimizing bone damage. To achieve the previously stated objectives, we first need to determine whether radiomics technology is sufficiently effective in diagnosing chronic osteomyelitis by assessing the combined area of lesions visible to the naked eye and their surrounding expanded regions (potential lesions).

Herein, we sought to develop an imaging-based osteomyelitis diagnosis model using radiomics analysis by comprehensively analyzing and extracting features from patient MRI data and evaluating its accuracy and reliability in determining the nature and extent of lesions. This approach could facilitate diagnosis and surgical decision-making, achieving a breakthrough in treating chronic osteomyelitis.

## 2 Materials and methods

### 2.1 Data collection

**FIGURE 1:**
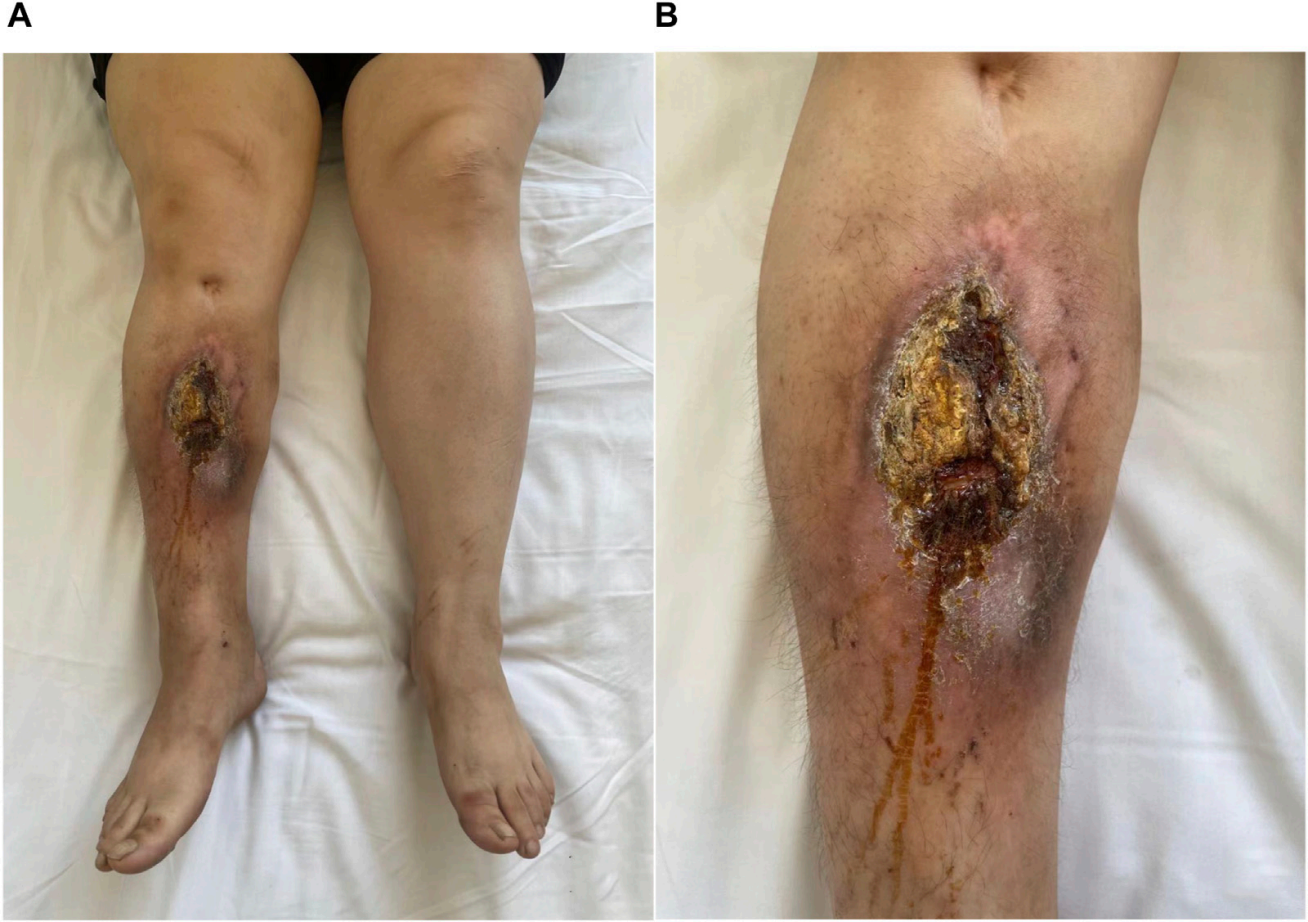
Macroscopic photo of a patient with chronic osteomyelitis.

A retrospective analysis was conducted on the clinical and imaging data of 93 patients with an initial diagnosis suspected to be chronic osteomyelitis of the long bones (Figure 1) who attended the First Affiliated Hospital of Xinjiang Medical University from January 2016 to May 2022. The study population comprised predominantly of males (n = 63/93, 67.7%), with a mean age of 35.5 (range: 19–67 years). Inclusion/exclusion criteria: Patients with a high suspicion of having chronic osteomyelitis of the long bones and requiring surgical intervention were included. These patients had well-established bone marrow infection persisting for more than 10 weeks, and the diagnosis was based on intraoperative histopathological examination or at least two sites with the same pathogen cultured or well-defined sinus tracts directly connected to the long bones, excluding cases with specific infections such as mycobacteria. The medical records of patients diagnosed with chronic osteomyelitis of the long bones included the following data: gender, age, anatomical site of infection, intraoperative microbiological culture results, treatment strategies, serum biomarkers, and MRI images after admission. Patients who were pregnant, breastfeeding, had metal implants, or were diagnosed with acute osteomyelitis, Charcot disease, diabetes, or chronic osteomyelitis in non-long bone locations were all excluded from the study. Moreover, if a patient had multiple medical records (multiple hospitalizations), only the most relevant record related to chronic osteomyelitis of the long bones was retained for analysis. Based on retrospective pathological analysis, the study ultimately included 48 patients with chronic osteomyelitis and 45 patients with non-chronic osteomyelitis. The Ethics Committee of The First Affiliated Hospital of Xinjiang Medical University approved the study with an informed consent exemption (K202308-11). Patients’ personal information was anonymized and de-identified prior to analysis.

### 2.2 MR scanning method

The patient image was acquired by a 1.5T MR scanner (SIEMENS). The following scanning parameters were used: T1WI TR 600 ms TE 9.5 ms; T2WI TR 3000 ms TE 88 ms; FS T2WI TR3600 ms TE 83 ms, FOV320 mm, matrix: 256 × 256. All patients underwent routine scanning, including T1-weighted sequences, T2-weighted sequences, and a T2-based short tau inversion recovery (STIR) sequence. Scans were performed in the coronal, sagittal, and axial planes according to the location of lesions, with a slice thickness of 4 mm and an interslice gap of 0.4 mm.

### 2.3 Lesion segmentation and radiomics feature extraction

#### 2.3.1 Image selection

MRI images of selected patients were extracted from the Picture Archiving and Communication Systems (PACS). The images were reviewed by a radiologist with over 10 years of musculoskeletal imaging diagnostic experience. T1-weighted images (T1WI) exhibited low signal intensity, whereas T2-weighted images (T2WI) and Short Tau Inversion Recovery images displayed high signal intensity for bone marrow inflammation. The presence of complete sequences without artifacts was confirmed before proceeding with image delineation.

#### 2.3.2 Lesion and perilesional area delineation

**FIGURE 2:**
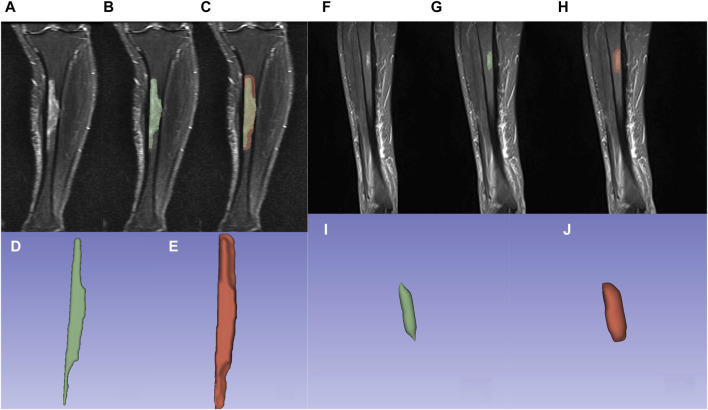
Osteomyelitis outlined using 3Dslicer. **(A,F)** Osteomyelitis in the proximal-medial tibia on the coronal view of the STIR sequence. **(B,G)** Osteomyelitis outlined within original ROI. **(C,H)** Osteomyelitis outlined within expanded ROI. **(D,I)** 3D model of osteomyelitis reconstructed from original ROI. **(E,J)** 3D model of osteomyelitis reconstructed from expanded ROI.

1) Lesion delineation was performed using 3D Slicer (version 5.3.0) software, simultaneously outlining both the outer contour and bounding box of the lesion. 2) Using the STIR sequence based on the MRI T2 sequence as a reference, the region of interest (ROI) was delineated in the coronal plane. 3) Manual delineation was conducted on each lesion plane while avoiding areas of necrosis and hemorrhage, with an emphasis on comprehensive coverage of the lesion substance. 4) Semi-automatic delineation was applied to the original region of interest (original ROI), displaying the lesion on the image, as well as the lesion area expanded by 5 mm (expanded ROI) from the original ROI. Subsequent manual adjustments were made to confirm the delineation scope, preventing any extension of the delineation beyond the bone structure. 5) For lesions with unclear borders, distinct high-signal areas were delineated. 6) In the case of multiple lesions, only the largest lesion was delineated. The results of lesion and perilesional area delineation are illustrated in Figure 2. 7) The delineation and review of the Regions of Interest (ROIs) were conducted by two radiologists, each boasting over a decade of expertise in musculoskeletal imaging diagnostics.

#### 2.3.3 Raiomics feature extraction

In this study, radiomics feature extraction was conducted using the pyradiomics module within the 3D Slicer software. A total of 1,037 features were automatically extracted for each ROI, encompassing various categories of features, including shape, firstorder, glcm, glszm, gldm, glrlm, and ngdtm. Furthermore, wavelet filtering and Laplacian of Gaussian (LoG) filtering techniques were applied. Wavelet filtering was applied to iteratively break down the initial image into various scales, thereby extracting valuable insights across diverse levels. In contrast, LoG filtering functioned as an edge enhancement filter, predominantly highlighting areas with significant variations in gray levels. By manipulating the sigma parameter in the LoG filtering process, we could regulate the prominence of texture characteristics. Smaller sigma values were employed to enhance intricate texture intricacies, while larger sigma values highlighted textural attributes on a broader scale (Lambin et al., 2012; van Griethuysen et al., 2017).

### 2.4 Statistical analysis

We used Python (ver 3.9.13) to process the two sets of radiomics features extracted from 3D Slicer, encompassing both the original and expanded ROI. Initially, data standardization was executed on the two datasets through a standardized method. The patient cohort was subsequently randomly partitioned into a training set and a testing set, maintaining a ratio of 7:3. Following this, dimensionality reduction was implemented on the extracted radiomics features via *t*-test, least absolute shrinkage and selection operator (LASSO) regression analysis, and the SelectKBest classifier.

The support vector machine (SVM) model was employed for modeling the kernel functions of both the original and expanded ROIs. The dimensionality-reduced features were employed for classification tasks. To assess the diagnostic efficacy of the established models, we conducted receiver operating characteristic (ROC) curve analysis, employing histopathological results as the reference standard. The model’s performance was evaluated by calculating the area under the curve (AUC), sensitivity, specificity, and accuracy. Discrepancies in the ROC between the two models were evaluated using the DeLong method (DeLong et al., 1988). All statistical analyses were carried out using Python (ver 3.9.13), and a significance threshold of *p* < 0.05 was employed to determine statistical significance.

## 3 Results

### 3.1 Radiomics feature extraction

Utilizing the pyradiomics module within the 3D Slicer software, feature extraction was conducted separately on the original ROI and the expanded ROI, yielding a total of 1,037 features. Following feature selection through t-tests and the LASSO method, 16 and 11 features were retained for the original and expanded ROI, respectively.

**FIGURE 3:**
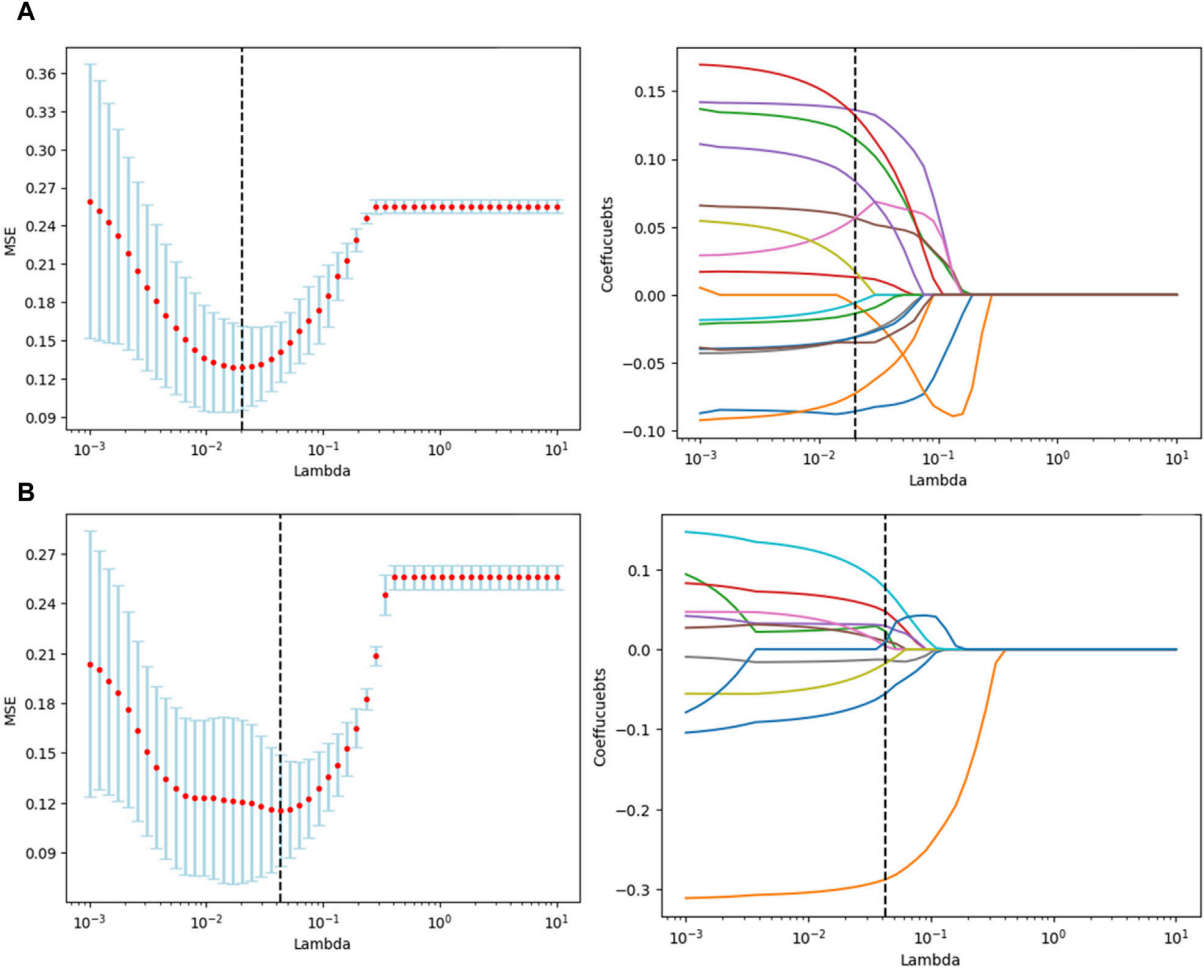
Texture feature selection using t-tests and selection operator (LASSO) for radiomics. **(A)** Original ROI texture feature selection. **(B)** Expanded ROI texture feature selection.

**FIGURE 4:**
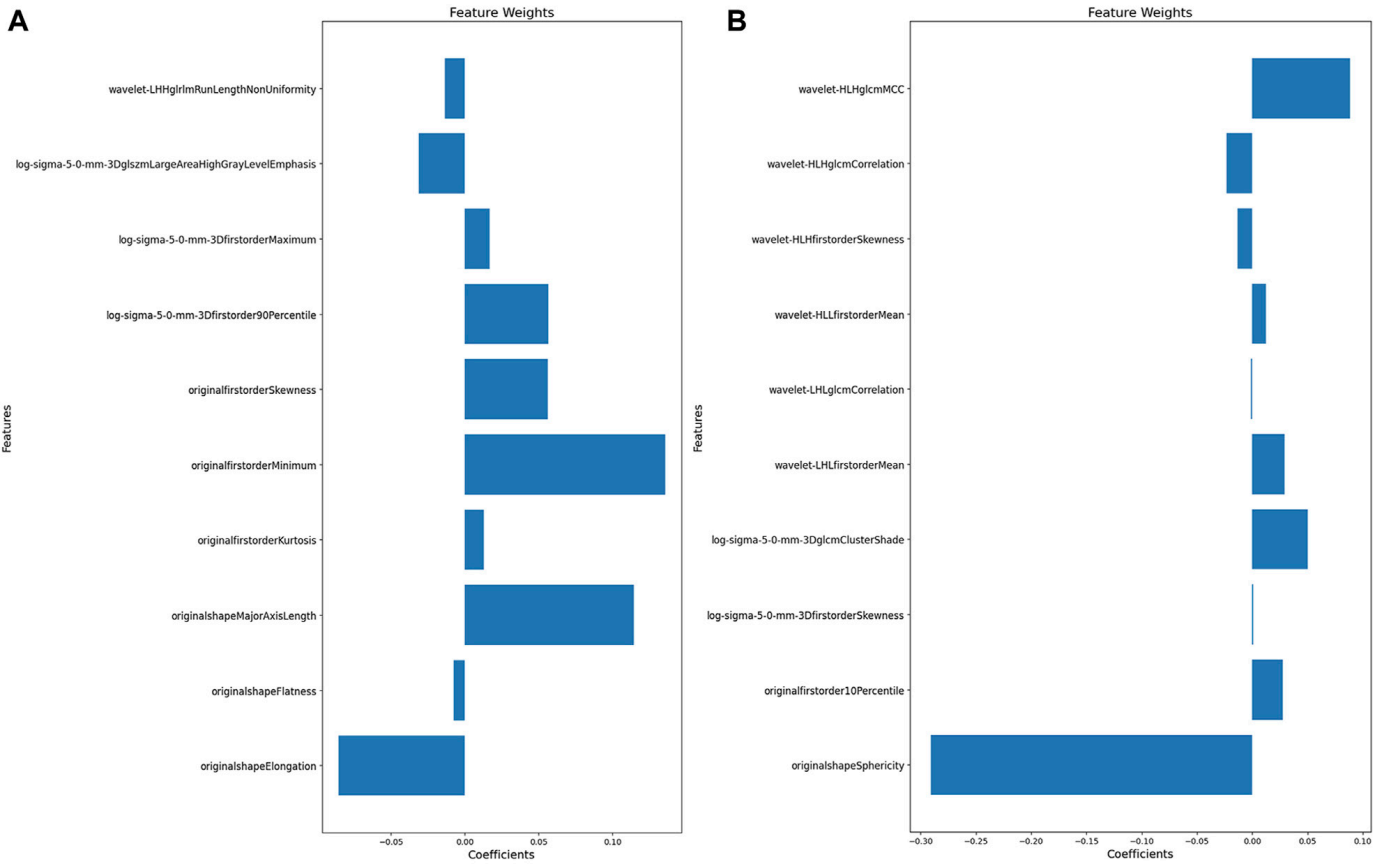
The features weight selected for original and expanded ROIs. **(A)** Features weight within the original ROI. **(B)** Features weight within the expanded ROI.

**FIGURE 5:**
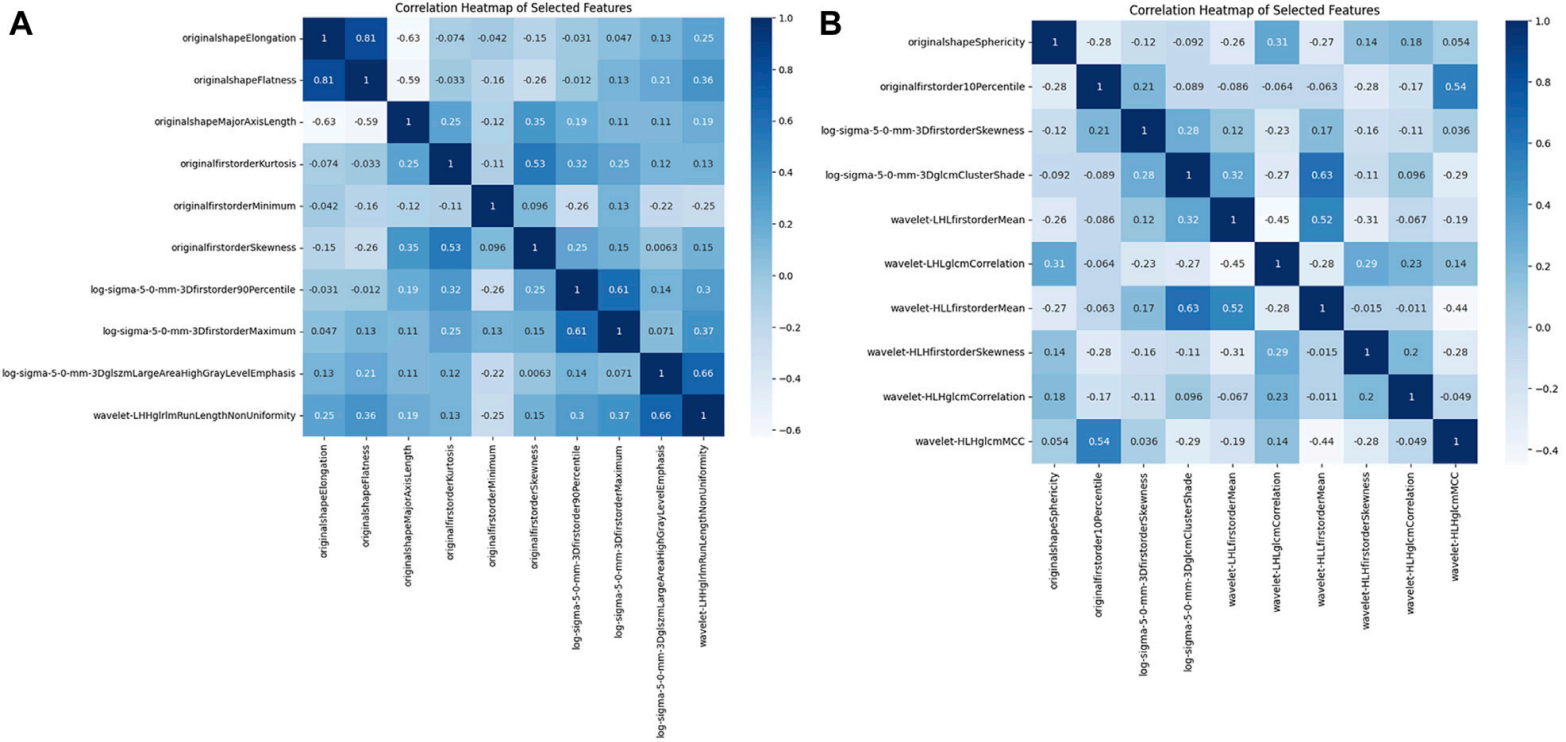
The correlation matrix heatmap. **(A)** Original ROI correlation matrix heat map. **(B)** Expanded ROI correlation matrix heat map.

Figures 3A,B illustrate the feature selection results, displaying the LASSO-driven selection of lesion texture features. To further reduce feature dimensionality and construct the SVM model, optimal polynomial degrees of 2 were determined during the hyperparameter grid search for SVM. The cost variables for the original ROI and the expanded ROI were set at 0.5 and 3.05, respectively, with scaling variables of 0.15 and 0.0625. Subsequent feature refinement was performed using the SelectKBest classifier, which employs statistical methods such as the chi-squared test, F-test, or mutual information to evaluate the relationship between each feature and the target variable. Features are ranked and selected based on the magnitude of the computed statistic (Bisong, 2019). By applying the SelectKBest classifier, we ultimately identified the top 10 features for the original and expanded ROI. Figures 4A,B depicts the feature weights selected for the original and expanded ROIs. The correlations between features within the original and expanded ROIs are visually represented through heatmap matrices in Figures 5A,B, respectively. All coefficients of total feature values were less than 0.7, implying the absence of collinearity among the features. The feature selection process underscores the significance of all gathered parameters as pivotal predictive elements for machine learning algorithms.

### 3.2 Comparison of model performance between original ROI and expanded ROI

**FIGURE 6:**
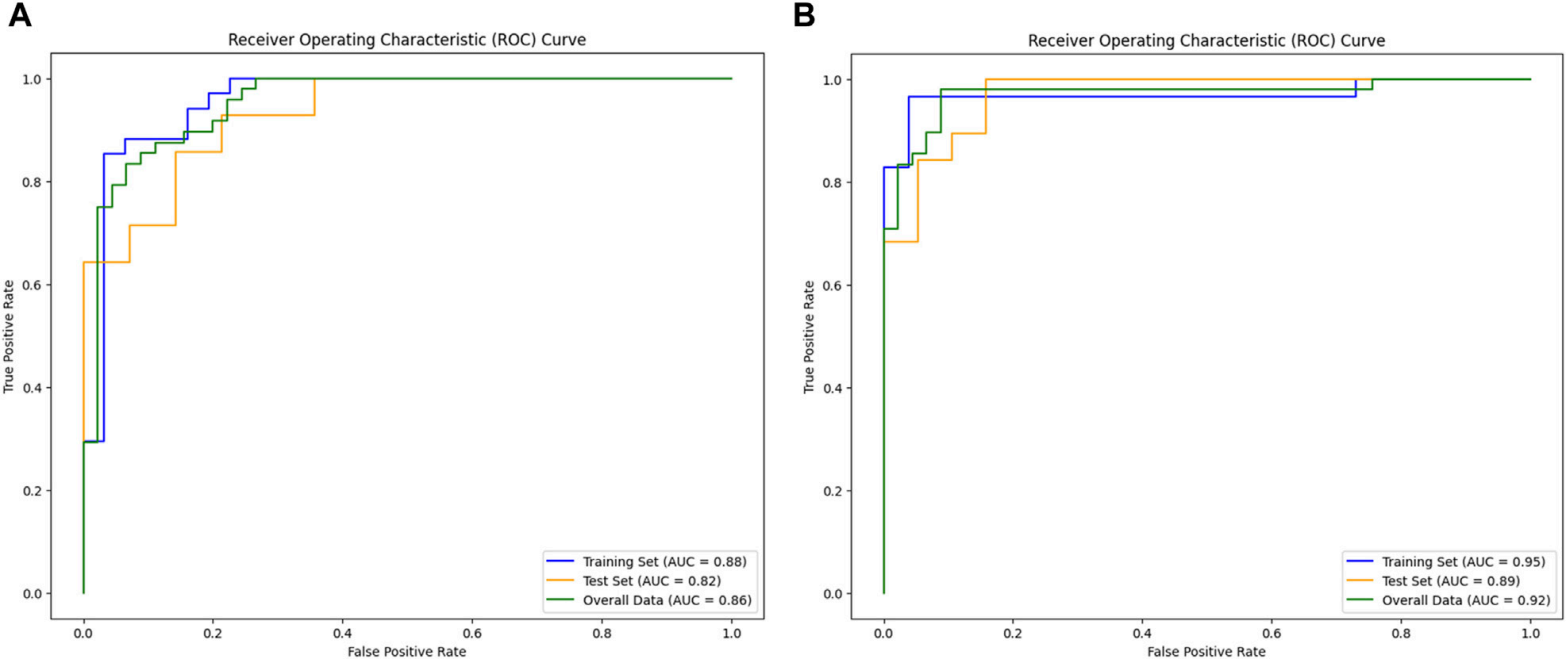
Comparison of model performance. **(A)** AUC of the original ROI. **(B)** AUC of the expanded ROI.

The original ROI model demonstrated an excellent diagnostic performance with an accuracy of 0.821, sensitivity of 0.857, and specificity of 0.785. Nonetheless, the expanded ROI model exhibited higher accuracy (0.894), sensitivity (0.947), and specificity (0.842), significantly outperforming the SVM model based on the original ROI radiomics features (Table 1). As depicted in Figures 6A,B, the expanded ROI model’s predictive performance was significantly superior to the original ROI (AUC value 0.920 vs. 0.859). DeLong’s test confirmed a significant difference between the two approaches (Z = 3.336, *p* < 0.001), indicating the enhanced diagnostic efficacy of the expanded ROI model.

**TABLE 1:** Diagnostic performance comparison between original ROI and expanded ROI models.

	Original ROI model	Expanded ROI model
Accuracy	0.821	0.894
Sensitivity	0.857	0.947
Specificity	0.785	0.842
AUC	0.859	0.920

## 4 Discussion

MRI stands out for its absence of electromagnetic radiation, swift examination times, and exceptional contrast depiction between bone and soft tissues. Beyond its capacity for multi-directional imaging, MRI boasts superior sensitivity in pinpointing lesion locations and extents compared to X-rays and CT scans (Hatzenbuehler and Pulling, 2011; Acikgoz and Averill, 2014). Furthermore, contrast-enhanced MRI can effectively delineate abscesses and sinus tracts related to chronic osteomyelitis, thus improving diagnostic precision. However, the utilization of gadolinium-based contrast agents presents iatrogenic hazards to patients. While MRI currently plays a pivotal role in osteomyelitis diagnosis, significant challenges remain for clinical diagnostics (Lee et al., 2016; Wong et al., 2019). In cases of chronic osteomyelitis, MRI reveals distinct patterns with low signal intensity on T1WI and high signal intensity on T2WI and STIR sequences. Additionally, the surrounding soft tissues often exhibit edema, inflammatory alterations, and localized osteolysis. However, distinguishing chronic osteomyelitis from conditions like osteosarcoma and osteotuberculosis which present very similar imaging features on MRI, can be challenging on plain MR scans. In this context, radiomics becomes particularly important since it can extract countless quantitative features from MRI images, dynamically observe lesions and their microenvironments in a non-invasive manner, discover a large amount of information hidden in MR image layers to predict clinical endpoints or lesion properties and provide possibilities for a comprehensive assessment of lesion heterogeneity (van Griethuysen et al., 2017; Muraoka et al., 2022), as well as providing more possibilities for precise guidance of the surgical scope. Studies have shown that the microenvironment around the lesion has important biological significance in terms of lesion growth, cell migration, inflammation, and other aspects (Lambin et al., 2012). However, most radiomics studies of osteomyelitis have focused on the lesion itself, and the perilesional area has not been comprehensively explored (DeLong et al., 1988). Integrating radiomics features extracted from the perilesional area could improve lesion predictive diagnostic efficiency and offers extensive prospects for precise surgical scope guidance by incorporating microtexture intricacies and multifaceted data.

Although previous studies have evaluated osteomyelitis using MRI (Oda et al., 2018; Iwasaki and Muraoka, 2020; Massel et al., 2021), there is limited research on the application of MRI radiomics in assessing chronic osteomyelitis. Therefore, further research and exploration in this field are valuable. In this study, we constructed two different regions of interest for osteomyelitis: the original ROI based on the lesion area of osteomyelitis and an expanded ROI expanding 5 mm beyond the original. Subsequently, two SVM models were developed using these ROIs to assess the diagnostic performance of MRI radiomics in osteomyelitis diagnosis. SVM, a supervised learning algorithm, endeavors to identify a hyperplane within the feature space that maximizes the classification of distinct classes of data points, thereby facilitating effective classification. In our study, SVM demonstrated effective applicability; MRI radiomics-based osteomyelitis diagnosis achieved enhanced sensitivity compared to previous studies on MRI’s diagnostic performance in osteomyelitis. For instance, compared to Hulsen et al.’s study (Hulsen et al., 2022), the sensitivity increased from 0.780 to 0.857, consistent with Hirotaka et al.’s findings regarding MRI radiomics’ robust diagnostic performance in pyogenic osteomyelitis (Muraoka et al., 2022). Expanding the original ROI by 5 mm resulted in the SVM model yielded marked enhancements in accuracy (0.894 vs. 0.821), sensitivity (0.947 vs. 0.857), specificity (0.842 vs. 0.785), and AUC value (0.920 vs. 0.859), attributed to the fact that not all osteomyelitis lesions manifest as high signal areas in MRI images, and subtle lesions beyond the high signal region might remain imperceptible to the naked eye. Importantly, the radiomics-based SVM model can identify these subtle features accurately, improving diagnotic accuracy. Our findings are consistent with the widely accepted perspective that eradicating osteomyelitis necessitates expanding the surgical scope (Lazzarini et al., 2002; McNally et al., 2022; Wu et al., 2023). Our results indicate that expanding the delineation range from 0 to 5 mm substantially enhanced MRI radiomics’ diagnostic performance in osteomyelitis, providing valuable insights for osteomyelitis surgical scope guidance.

Differentiating healthy tissue from non-viable tissue during early-stage surgery is inherently complex. Therefore, recognizing the significance of early, thorough debridement is widely acknowledged (Li et al., 2020). Eckardt et al. initially proposed aggressive debridement, akin to managing giant cell bone tumors, for chronic osteomyelitis treatment (Eckardt et al., 1994). Some scholars even advocate treating it like malignancy (Simpson et al., 2001), consistent with our findings. Thus, applying radiomics techniques to accurately determine lesion extent holds significant practical benefits for patients, impacting short-term surgical outcomes and long-term disease control and prognosis.

After applying t-tests and LASSO-based feature extraction, the original ROI model retained 16 features, while the expanded ROI model retained 11. The potential drawbacks of excessive features, including increased false discovery rates, overfitting, and diminished model generalization efficacy, have been highlighted (Kumar et al., 2012; Gillies et al., 2015). To mitigate these risks and enhance model accuracy, the SelectKBest classifier was employed, further reducing feature dimensionality to 10 for both models.

Based on our feature selection, the original ROI model highlighted Elongation, Major Axis Length in the shape features, and the Minimum feature in the first-order statistical features as optimal descriptors for characterizing the texture attributes within the osteomyelitis region. In contrast, within the expanded ROI model, the wavelet filter’s maximum correlation coefficient (MCC) feature and sphericity from the shape-related feature set emerged as the most informative in delineating the textural characteristics of the expanded osteomyelitis suspicious area. In the original ROI model, Elongation and Major Axis Length encapsulate the primary directional characteristics of the region of interest’s shape and the length of its primary axis within the enclosed ellipsoid. Elongation assesses the extent to which the ROI’s shape appears elongated, while Major Axis Length quantifies the principal dimension of the ROI. During osteomyelitis imaging, a lesion can lead to anomalous enlargement and morphological alterations within the adjacent bone marrow architecture. This often manifests as an elongated lesion region with an irregularly expanded shape, collectively indicating the structural attributes of the bone and the extent of osteomyelitis infiltration (He et al., 2021). Utilizing the Minimum feature within the first-order statistical features primarily assesses the minimum grayscale intensity within the lesion region. In cases where the original ROI exclusively encompasses the osteomyelitis area displaying high signal intensity on the MRI image, the overall image’s minimum grayscale value tends to be elevated. Conversely, the expanded ROI’s delineation encompasses regions outside the lesion, often represented by darker areas on the MRI image. This inclusion leads to a comparatively lower overall minimum grayscale value, resulting in the reduced significance of the Minimum feature. Meanwhile, sphericity quantifies an object’s resemblance to a perfect sphere, with lower values indicating deviation towards irregularity. In the context of the expanded ROI model, sphericity emerges as a pivotal discriminatory feature, reflecting the extent of roundness within the lesion area in relation to a spherical shape (Priya et al., 2021) Its prominently negative weight might stem from the indistinct boundaries and unevenness of the expanded ROI, resulting in a modified shape. Additionally, the MCC feature linked to the wavelet filter is a gray-level co-occurrence matrix (GLCM) component, signifying the intricacy of texture patterns (Su et al., 2023). Given the broader delineation of regions within the expanded ROI model, implying heightened textural complexity compared to the original ROI, the MCC feature was more prominent in the expanded ROI model.

Across the original and expanded ROI models, the shape feature retained a notably substantial weight ratio among all the screened features. Previous studies have effectively employed shape features in delineating tumor aggressiveness (Limkin et al., 2019). Similar to the invasive characteristics exhibited by tumors, osteomyelitis also demonstrates comparable aggressiveness. Shape features possess a unique capability in assessing bone erosion, which can effectively differentiate chronic osteomyelitis from other conditions in magnetic resonance imaging.

The application of radiomics ensures a relatively high level of accuracy in differentiating between residual lesions in chronic osteomyelitis, such as infected tissue, inflammatory tissue, and edema, despite their similar high signal intensity on imaging. Implementing an expanded lesion detection strategy can be likened to providing young doctors with a vantage point on the accomplishments of experts, reflecting the inevitable trajectory of rapid artificial intelligence advancement. However, it should be borne in mind that despite the immense potential of radiomics technology, its effective utilization still hinges on the expertise and experience of medical professionals for thorough analysis and interpretation. Radiomics technology functions as a supplementary tool, and doctors must still integrate elements like clinical history, physical examinations, and other supplementary test outcomes to arrive at the ultimate diagnosis and treatment decisions.

Our study has several limitations. Firstly, it is a retrospective study, potentially subject to information bias. Secondly, our discussion solely pertains to the impact of MRI radiomics on the diagnostic efficacy of osteomyelitis. Thirdly, comparing radiomics features with imaging morphological characteristics is necessary. In our future research, we plan to construct a fusion model based on both radiomics and morphological features to further explore and validate their combined diagnostic value. However, previous studies have shown that certain clinical factors, such as a history of previous wounds (Lavery et al., 2009) and microbial infections (Bury et al., 2021), may also influence the qualitative diagnosis of osteomyelitis. Therefore, in future research, integrating clinical features with radiomics may become an expanded focus to provide a more comprehensive disease diagnosis and treatment guidance. Additionally, further research is needed to investigate the extent of expanded delineation in conjunction with radiomics, aiming to achieve optimal accuracy, sensitivity, specificity, and other information. We also plan to explore additional radiomics models, such as random forest models and logistic models, to compare the diagnostic performance of different models and identify the most suitable radiomics model for osteomyelitis diagnosis.

## 5 Conclusion

MRI radiomics-based methods yielded promising results for diagnosing chronic osteomyelitis, especially when utilizing an expanded ROI model that enhances diagnostic accuracy. With further validation from larger-scale, high-quality studies in the future, this approach can potentially become a valuable tool for guiding surgical interventions in chronic osteomyelitis, providing accurate diagnosis and precise localization of the affected lesion areas, ultimately optimizing surgical decision-making and improving patient outcomes.

## Data Availability

The raw data supporting the conclusion of this article will be made available by the authors, without undue reservation.

## Abbreviations

ROI, Region of Interest; STIR, short tau inversion recovery; PACS, Picture Archiving and Communication Systems; T1WI, T1-weighted images; T2WI, T2-weighted images; LoG, Laplacian of Gaussian; SVM, support vector machine; ROC, receiver operating characteristic; AUC, area under the curve; MCC, maximum correlation coefficient; GLCM, gray-level co-occurrence matrix.
